# Australian Defence Force Centre for Mental Health Second Opinion Clinic – first ten years of operation

**DOI:** 10.1177/10398562231190780

**Published:** 2023-07-27

**Authors:** Duncan Wallace, Carla Meurk, Andrew Moss, Ed Heffernan

**Affiliations:** ADF Centre for Mental Health, Joint Health Command, 7800 Australian Defence Force; and School of Psychiatry, University of New South Wales, Sydney, NSW, Australia; Forensic Mental Health Group, 359385 Queensland Centre for Mental Health Research; and School of Public Health, Faculty of Medicine, The University of Queensland, Brisbane, QLD, Australia; Joint Health Command, Australian Defence Force, Canberra, ACT, Australia; ADF Centre for Mental Health, Joint Health Command, Australian Defence Force; Forensic Mental Health Group, Queensland Centre for Mental Health Research; and School of Public Health, Faculty of Medicine, The University of Queensland, Brisbane, QLD, Australia

**Keywords:** Military mental health, second opinion, tertiary referral clinic, telepsychiatry

## Abstract

**Objectives:**

The aim is to report the operation of the Australian Defence Force Centre for Mental Health (ADFCMH) Second Opinion Clinic (SOC) after its first 10 years of operation.

**Method:**

Demographic data and clinical data were recorded and analysed for all Australian Defence Force (ADF) personnel (*n*=209) seen at the clinic from 2011 to 2021.

**Results:**

Assessment at the clinic led to a change in diagnosis from that given at the time of referral in 40.7% (*n*=85) of members seen. Of the total members assessed at the SOC, 55.9% (*n*=117) had been on an at least one operational deployment. Mood disorders were the most common mental disorders seen among personnel at the SOC.

**Conclusions:**

The ADFCMH SOC is a valuable clinical resource supporting ADF health services nationally and provides an example of an effective mental health tertiary referral service.

The mental health of Australian Defence Force (ADF) personnel is a priority^1,2^ with ongoing concerns about military and veterans’ suicide leading to the establishment of the Royal Commission into Defence and Veteran Suicide in 2021. One of the challenges in providing mental health assessment and care to ADF members is the need to combine an understanding of the unique environment, stresses and culture of the ADF and how this interacts with mental health presentations and functional capacity.^3^

The ADF Centre for Mental Health (ADFCMH) was established in 2010 as part of the Australian Government’s response to the 2009 Dunt Review of Mental Health Services in the ADF.^4^ The review also recommended that the Centre provide expert clinical advice and assessment services for complex mental health cases across the ADF leading to the development of the Second Opinion Clinic (SOC) in 2011. The SOC provides a tertiary referral service delivered by psychiatrists and psychologists with knowledge and experience of the ADF who undertake comprehensive mental health assessments of ADF personnel and provide diagnostic, management and service fitness opinions for the member’s treating mental health team. Ongoing care or follow-up of members seen is not provided by the SOC.

The ADFCMH SOC is a unique service offered to members irrespective of their location. Those referred usually have severe, complex or difficult to treat mental disorders with significant comorbidity. This article describes the findings of an audit of the first 200 patients referred to the ADFCMH SOC across its first 10 years of operation, highlighting the types of serious mental illness that can impact on service personnel.

## Objectives

The aim is to describe the demographic, clinical and service characteristics of the first 200 patients of the ADFCMH SOC across a 10-year period.

## Methods

### Service context

Uniformed and civilian Defence Health Service medical officers across Australia can refer members of the ADF to the SOC using a standardised referral form that is reviewed by the ADFCMH psychiatrist. Patients must be serving members of the ADF who have been previously assessed by at least one psychiatrist. Assessment is jointly performed by both a psychiatrist and psychologist to provide a comprehensive, multi-disciplinary assessment. The ADFCMH has a pool of psychiatrists and psychologists with varied sub-speciality expertise enabling a decision about the most appropriate specialist to undertake the assessment. Psychiatrists and psychologists are usually either full-time or part-time (reserve) members of the ADF.

Assessment begins with a document review which includes the member’s psychology file and medical records, including their recruit medical examination and psychology assessment. Referred members are asked to provide copies of their last two annual performance appraisal reports, to provide objective evidence of their work performance.

Members referred to the SOC may be asked to complete a range of psychological screening tests prior to the assessment (see Box 1). A comprehensive clinical interview is undertaken, the duration of which is usually about 2 hours. Relevant collateral history is obtained, with the member’s consent. Diagnostic decision making is by clinician consensus using the Diagnostic and Statistical Manual of Mental Disorders, DSM-IV TR^5^ from 2011 to 2013, then DSM-5.^6^

**Box 1:** Commonly used psychological screening tools at the Second Opinion Clinic

Kessler Psychological Distress Scale (K10).Depression Anxiety Stress Scale (DASS-21).Beck Depression Inventory (BDI-II).Dimensions of Anger Reactions (DAR-5).Alcohol Screen (AUDIT).Post-traumatic Stress Disorder Check List (PCL-5).

A confirmed diagnosis of PTSD was made by the assessors when clinical symptoms were considered to have met diagnostic criteria from previous clinical reports and/or at the time of assessment sometimes with supporting evidence from PTSD Checklists (PCL-C; PCL-5) and Clinician Administered PTSD Scale (CAPS-IV and CAPS-5) assessments. Findings of sub-syndromal PTSD/adjustment disorder or probable PTSD were made when it was considered that the member had significant symptoms and some impairment but did not reach full diagnostic criteria.

Reports include clinical data, diagnostic opinions and management recommendations including pharmacotherapy, psychotherapy, additional specialist input (e.g. addiction medicine) and advice about fitness for duty, sea service, deployment and capacity for retention in the ADF where indicated.

### Data collection

A database of assessments was maintained to enable aggregated reporting of SOC activities to command. The database contains details of members’ age, rank, service, diagnosis, whether the given diagnosis was changed as a result of the assessment, whether the member had deployed and whether they were seen in person or by video-conferencing. This data has been collected for all assessments conducted between 2011 and 2021.

## Results

As of 14 December 2021, a total of 209 ADF personnel were consecutively referred and assessed at the SOC. The reasons for referral are recorded in Table 1. There are a greater number of reasons for referral than individuals seen due to multiple reasons for referral for some members.

**Table 1.** Reasons for referral of Australian Defence Force members to the Second Opinion Clinic

Reason for referral	% (*n*) *N* = 209
Confirmation/review of a current diagnosis	77 (160)
Advice on medical management	59 (123)
Assessment of fitness for retention in the Australian Defence Force	33 (69)
Advice on medical classification within the Australian Defence Force	5 (10)
Fitness for Australian Defence Force operational deployments	9 (19)
Fitness for specific Australian Defence Force postings/transfer of employment category	3 (6)

Demographic details are displayed in Table 2.

**Table 2.** Demographic characteristics of members seen at Australian Defence Force Centre for Mental Health Second Opinion Clinic between 2011 and 2021, *N* = 209

	Numbers	%
Age (mean)	Male 33.8	75.1%
	Female 30.3	24.9%
**Age group**		
18–27	68	32.5
28–37	72	34.4
38–47	50	23.9
^a^48–57/58+	19	9.1
**Rank**		
OFFR	62	29.7
Non-commissioned officer^b^	67	32.0
Other ranks	80	38.3
**Service**		
Navy	64	30.6
Army	118	56.4
Air force	27	12.9

^a^Age groups merged to prevent statistical disclosure control issues.

^b^Non-commissioned officers (NCOs) include Leading Seaman/CPL rank and above.

### Telepsychiatry

Telepsychiatry was used by the SOC from its inception in 2011,^7^ with 40.7% (*n*=85) of the referrals seen by this modality from 2011 to 2021. There were 161 members assessed by the SOC before the declaration of COVID-19 pandemic, 11 March 2020, with 50 (31%) seen by video-conferencing. During the COVID-19 pandemic, from 11 March 2020 to 14 December 2021, 72.9% (35 out of 48 members) seen were by video-conferencing.

### Diagnosis

Diagnoses made by the SOC are listed in Table 3. Mood disorders were the most common mental disorders, including major depression and adjustment disorders with depressed mood (53.1%), and bipolar disorder (9%). Substance use disorders (27.3%), trauma and stressor-related disorders (23.4%) and personality disorders (17.7%) were also common diagnoses. There was a small number of psychotic disorders, neurodevelopmental disorders, anxiety disorders, neurological disorders and eating disorders diagnosed.

**Table 3.** Psychiatric diagnoses made at the Second Opinion Clinic, Australian Defence Force centre for mental health between 2011 and 2021

	Total number of diagnoses	Percentage of patients seen (%)
**Depressive disorders**	111	**53.1**
Major depressive disorder	83	39.7
Adjustment disorder with depressed mood	22	10.5
Depressive disorder NOS	2	1.0
Dysthymic disorder	1	0.5
**Substance use disorders**	57	**27.3**
Alcohol use disorder	47	22.5
Other SUD	10	4.8
Gambling disorder	4	1.9
**Trauma and stressor-related disorders**	50	**23.9**
Post-traumatic stress disorder	38	18.2
Probable PTSD	6	2.8
Sub-syndromal PTSD/adjustment disorder (sub-syndromal PTSD)	6	2.8
**Bipolar disorder**	19	**9**
Bipolar II disorder	14	6.7
Bipolar I disorder	5	2.4
**Personality disorders**	37	**17.7**
**Psychosis**	10	**4.8**
Schizophreniform disorder	5	2.4
Schizophrenia/brief psychotic disorder/unspecified schizophrenia spectrum	5	2.4
Disorder/residual schizophrenia		
**Neurodevelopmental disorders**	10	**4.8**
Attention deficit hyperactivity disorder/autism spectrum disorder		
**Anxiety disorders**	10	**4.8**
Obsessive compulsive disorder	6	2.9
Panic disorder	4	1.9
**Eating disorders**	7	**3.3**
**No current mental disorder**	19	**4.3**
**Neurological disorders**	7	**3.3**

(*N*.B. Due to the presence of comorbidities, the total *n* (%) will be more than 100%).

A significant proportion of members seen were diagnosed with multiple mental disorders (44%, *n*=92), nearly a quarter (24%, *n*=51) had two diagnoses, and (20%, *n*=41) had three or more diagnoses.

Of note, 43% (*n*=20) of members seen with alcohol use disorder were recommended to be referred to an addiction medicine specialist.

### Change in diagnosis from clinic assessment

Assessment at the SOC led to a change in diagnosis from that given at the time of referral in 40.7% (*n*=85) of members seen. Examples of changes in diagnoses included single episode of major depressive disorder to recurrent major depression; unipolar depression to bipolar disorder; and bipolar disorder to borderline personality disorder or no current mental disorder.

### Deployment

Just over half (55.9%, *n*=117) of total members assessed at the SOC had been on an at least one operational deployment. Of those who had been on at least one operational deployment, 33.3% (*n*=39) received a diagnosis of a trauma-related disorder. 22% (*n*=11) of all 50 members diagnosed with trauma-related disorders had not been on any operational deployment.

## Discussion

The ADFCMH SOC provides a useful national mental health service to support the mental health care of ADF personnel. The strengths of the clinic include the professional and military cultural expertise of the assessing staff; the detailed reassessment of clinical records – a process that may be impractical for the referrer and other treating clinicians; and having two assessors allowing differing perspectives. These are features which have been recognised for their importance in tertiary services and clinical decision making.^8^

Patients seen at the clinic met a number of criteria proposed for assessment at tertiary services for complex or refractory disorders including diagnostic uncertainty hampering treatment, persistently high symptom burden, significant impact on functioning, persisting patterns of incapacity despite appropriate treatment and multiple comorbidities increasing the likelihood of chronicity.^9,10^

The most frequent reason for referral to the SOC was to review diagnosis. Following assessment, there was a large proportion of members whose diagnoses were changed (40.7%). This is important as it was likely to have resulted in changes in treatment and also potentially fitness for duty or retention in the military. This figure was slightly lower than that described at the SOC in 2015 when 50% of members seen (*n*=58) received a different diagnosis.^7^ Due to the limited literature on tertiary referral mental health clinics, it was not possible to make comparisons about rates of diagnostic change.^11,12^

When the SOC was established, its scope was limited to assessment, providing treatment advice and recommendations on fitness for service. This prevented the follow-up of members seen to determine whether recommendations were accepted and what outcomes were achieved. While this is a constraint of tertiary services more generally, it is recognised that the ability to access more appropriate treatment would have been a significant outcome for the individuals concerned.

The number of ADF personnel seen at the SOC with a diagnosis of alcohol use disorder who had not previously been referred to a medical specialist in this field (43%; *n*=20) was concerning and suggested a lower-than-expected rate of referral to addiction medicine physicians and psychiatrists across the ADF. This finding recently led to the establishment of a pilot addiction medicine specialist project to raise awareness among health services personnel about the speciality and to promote referrals.

Psychological screening test results were used as an adjunct to clinical assessment by staff at the SOC. Experienced military mental health personnel at the Clinic were mindful that military personnel are less likely to report symptoms of mental disorder in identifiable health screens compared to anonymous surveys^13^ because of concerns about potential adverse career effects of a mental health diagnosis.

Finally, this study was limited by the naturalistic design that meant that case selection and clinical outcomes were based on cross-sectional expert opinions of consecutive cases. These are considered to be understandable challenges associated with research based on clinical services.

## Conclusion

The ADFCMH Second Opinion Clinic is a valuable mental health capability that provides practical expert clinical advice to support members of the ADF with complex mental disorders.
